# The Spanish population’s interest in climate change based on Internet searches

**DOI:** 10.1057/s41599-023-01736-5

**Published:** 2023-05-12

**Authors:** Olaya Álvarez-García, Jaume Sureda-Negre, Rubén Comas-Forgas, Miquel F. Oliver-Trobat

**Affiliations:** Department of Applied Pedagogy and Educational Psychology, University of the Balearic Islands, Palma, Balearic Islands Spain

**Keywords:** Cultural and media studies, Cultural and media studies

## Abstract

The climate crisis is one of the most important global problems facing humanity. Analyzing the search for information on climate change (CC) on the internet can be a predictor of public interest in this problem and, therefore, of the degree of concern exhibited by citizens. This study analyzes the interest in CC among the Spanish population and identifies some variables that may influence this interest. The methodology involves the collection and analysis of data obtained from SEMrush and Google Analytics. We analyzed the search trends of four key descriptors related to CC (“climate change,” “global warming,” “climate emergency” and “greenhouse effect”) during two periods of time, and the relationship between these searches and three relational variables (volume of news in the media, occurrence of extreme weather events and CC-related events). The results indicate that the Spanish population’s interest in CC via the Internet has increased in recent years and is directly influenced by variables such as media coverage of CC, events related to CC, and social pressure exerted by social movements for CC. Some proposals are discussed and presented in relation to the concern for this problem.

## Introduction

The scientific evidence on climate change (CC) and its consequences has been highlighted in numerous reports released by the Intergovernmental Panel on Climate Change (IPCC, 2018, 2019a, 2019b, 2023). The subject has also been debated and discussed in different international forums (Conferences of Parties, COP), with the Paris Agreement of 2015 being the greatest milestone in terms of limiting greenhouse gas emissions and fighting CC worldwide. This international treaty is legally binding, and the members, after its ratification, had to propose different strategies, plans, programs, and regulations through which they would limit global warming to 1.5 °C based on preindustrial levels (United Nations, 2015).

For the achievement of the proposed goal, public pressure is essential (Phillis et al., 2013). Bold decisions that the climate situation requires are difficult to assume without a citizenry aware of the consequences of not making such decisions. Hence, it is of great importance to explore the interest of the population in environmental issues in general and the climate crisis in particular. In this sense, there have been recent investigations that address these issues: Eurobarometer (European Commission, 2019, 2020, 2021), reports released by the Pew Research Center (Pousther and Huang, 2019; Tyson and Kennedy, 2020; Tyson et al., 2021) and studies by the Yale Program on Climate Change (Leiserowitz et al., 2021a, 2021b). These surveys established, among other things, that in 2021, a 18% of European citizens positioned CC as “the single most serious problem facing the world*”* just ahead of other problems commonly reported by citizens, such as poverty, hunger, and lack of clean water (17%) or the spread of infectious diseases (17%) (European Commission, 2021) and that 64% of the American population believe that reducing the effects of CC should be a “top priority to ensure a sustainable planet for future generations, even if that means fewer resources for addressing other important problems today” (Tyson et al., 2021).

To understand public opinion and the informative interests of citizens regarding CC approaches such as the analysis of data derived from information searches on the subject on the internet have been used. These data are primary indications of the interests, opinions, and doubts of the population about CC. This approach is particularly relevant because, for nearly half of the European population, the Internet and social networks are the main sources of information regarding the environment (European Commission, 2020).

Studies based on metrics derived from internet searches have become an effective approach to track citizen interest in various topics, being, in some cases, even more useful than traditional surveys (Ripberger, 2011; Vosen and Schmidt, 2011). *Google Trends* has become the most used tool for this type of approach (Vosen and Schmidt 2011; Mellon, 2014), whose advantages include access to a large sample at a low cost, guaranteed anonymity, and analyses of long periods of time (Zhu et al., 2012; Mellon, 2014, Orduña-Malea and Aguillo, 2015).

Some researchers have used *Google Trends* to understand the interest of the population in issues related to biodiversity conservation (Ficetola, 2013; McCallum and Bury, 2013, Proulx et al., 2014; Burivalova et al., 2018; Zieger and Springer, 2021; Nghiem et al., 2016) and various aspects of CC. An increase in internet searches on the subject coinciding with international COP climate meetings and their media coverage has been reported (Hartwell et al., 2020). Likewise, the positive influence of various celebrities (Leonardo Di Caprio, for example), documentaries on CC (*Before the Flood*, for example), and the climatic protests of the student movement *Fridays for the future* (Mavrodieva et al., 2019) on public interest in the issue has also been analyzed and described. Other studies suggest that extreme weather events also have a positive effect on internet search behavior in relation to CC (Lang, 2014; Lang and Ryder, 2016). Signs of concern shown by elites, media coverage and, to a lesser extent, public access to accurate scientific information are also influential factors related to public concern regarding CC (Brulle et al., 2012).

This study addresses the issue of public interest in the CC phenomenon, so as to provide evidence regarding the interest about this issue among the Spanish population. The objectives of this study are as follows:

1. Identify, analyze, and classify the descriptors used in organic searches on the internet conducted in Spain about CC between April 2020 and March 2021;

2. Analyze the volume of activity and internet search trends for descriptors related to CC in Spain between April 2020 and March 2021;

3. Compare search trends for and interest in CC in Spain for the period between April 2016 and March 2021; and

4. Analyze the relationship between public interest on issues related to CC in Spain and various variables: (a) volume of news on the subject in the media; (b) extreme weather events; and (c) CC-related events of great importance (for example, world summits on climate, demonstrations, and strikes).

This work focuses on the Spanish context, and the proposed objectives are more relevant if one takes into account that in 2019 the public debate on CC was especially prolific in Spain. Climate-related student movements (Youth for Climate^1^) and homologous movements among teachers (*Teachers for Future España*^2^) and among mothers (*Mothers by climate*^3^) put the climate emergency at the center of the debate and probably accelerated the promulgation of climate emergency declarations in some of the Autonomous Communities (regions in which Spain is administratively divided) as well as at the national level. In all cases, these are government commitment agreements for the design of actions to address the climate crisis. Furthermore, the celebration of COP25 in Madrid between December 2 and 13, 2019, could have increased the interest in CC among citizens. Additionally, in some Autonomous Communities, this debate has already been initiated with the development of regional climate laws (for example, the law of Catalonia or the Balearic Islands), and the process to approve a state law on CC and energy transition has also begun.

In 2019, the vast majority of the Spanish population (89%) considered CC to be a “very serious” problem, an increase of three percentage points compared with that in 2017 (European Commission, 2019). Material damage and economic losses caused by extreme weather events (in Spain, the storms Odette or Filomena and the storm Gloria) could influence this perception. Similarly, phenomena such as increases in episodes of high temperatures or heat waves, which cause more deaths in Spain than other natural disasters, could also influence the phenomenon (Government of Spain, 2021). More recent data (Ideara, 2021) indicate that 93.5% of the Spanish population considers CC to be real and that 73.3% believe that CC is being given less importance than needed. In fact, this concern, due to the low importance given to it, has increased by 16.1 percentage points since 2012 (Meira et al., 2013).

As previously shown, the search for information on CC on the internet is a predictor of public interest in this problem and, therefore, of the degree of concern of the public. An analysis that focuses on internet searches on CC in Spain was found in one study (Fernández-Reyes, 2015). Other studies adopted an approach that focuses more on assessing the quality of the information disseminated by the media than on considering the interest that the population may have in seeking information on this problem (Fernández-Reyes and Mancinas-Chávez, 2013; Pérez and Perales, 2018). In this context, identifying interest in CC as well as the variables that may influence it can improve the level of knowledge and evidence regarding the degree of awareness of CC among the Spanish population. In addition, it could also provide guidance on how the effectiveness of proposed actions to raise awareness can be improved. Additionally, an increase in citizen concern for CC would entail greater social pressure, which could influence political actors. To this end, authors such as Miléř and Sládek (2011), point to the importance of basic climate literacy to drive this engagement and public pressure, so that policies for climate action can be developed. This literacy aims to understand climate science in order to make informed decisions that improve our quality of life (Dupigny-Giroux, 2010) and aim to help the public better understand the climate system (Damico et al., 2018).

## Method

This study involves the collection and analysis of two sets of data from searches carried out on the internet in Spain on CC. First, the “Keyword magic tool” function of *SEMrush* was used to generate, from keywords and descriptors, a list of related keywords used for organic searches in *Google*. *SEMrush* is an online marketing and visibility analysis tool that uses *Google Analytics* data and allows examining, among other aspects, the grouping, management, and traffic generated by keywords. Following a previous study by Nanda et al. (2021) these keywords are the search terms used as a search engine in Google and are then used to list websites in the results pages that are returned. Data analysis is based on the Google search engine which is more precise and has better quality than any other search engine (Kostagiolas et al., 2021). From SEMrush we used the following features: (1) Keyword Search Volume: the average number of monthly searches of a particular keyword over 12 months and (2) Keyword Trend: the level of interest in a search query over 12 months.

For this study, an initial search of concepts related to CC was applied, considering the keywords “climate change,” “global warming,” “climate emergency” and “greenhouse effect.” Notably, the terms “greenhouse effect” and “climate change” are not scientifically synonymous because the greenhouse effect is a phenomenon that occurs naturally in the atmosphere; an increase in the greenhouse effect is the cause of global warming, and the consequence is CC. However, they are terms that are used interchangeably by the media and the general public when referring to this problem (Caballero et al., 2007). These misconceptions were already identified among citizens in the 1990s (Kempton, 1991; Löfstedt, 1991) and persist nowadays (Leiserowitz et al., 2010; Crosman et al., 2019). Unfortunately, are part of the common heritage of CC.

Thus, the consideration and inclusion of “greenhouse effect” among the terms analyzed are relevant for this study. From these initial searches, a first list of 55,280 keywords was obtained for searches carried out in Spain on CC. Given the large volume of keywords located in the first search, something intrinsic to this methodological approach as stated by Lillo and Ruggieri (2021) is the impossibility of counting all queries, especially when dealing with a long distribution queue; therefore, to set up a cut-off point only the terms that had monthly organic search averages equal to or >100 were considered. This cut-off point is quite significant as represents 87% of the total searches performed in Spain for the keywords considered during the period analyzed. The four keyword lists with 100 or more monthly searches were downloaded as a single search. An Excel spreadsheet was created with the following data from *SEMrush* using the “Keyword magic tool” option: (1) monthly volume of searches in *Google* for each keyword (provides the average monthly searches using a given keyword in a 12-month period, in our case from April 2020 to March 2021); and (2) trend data that measure the interest in a specified keyword during a 12-month period (the metric is based on the changes in the number of queries per month).

Once all the keywords were downloaded in a single data matrix in Excel, the descriptors that were duplicates and keywords that had no relationship with the organic searches clearly associated with CC were eliminated (for example, “Trump climate change”, “climate change girl”, “Bill Gates climate change”, and “polar bear global warming”). The final list of 200 keywords comprised the final sample for the first dataset of the study. All 200 keywords were classified into categories based on the purpose of the search. To do so, one of the authors analyzed the 200 terms and generated initially six categories with them and submitted this to the rest of the authors that had to review the initial clustering generated. After a triangulation process, the other authors generated two new categories and a consensus was achieved and a set of 8 categories were generated to classify the sample constituted by the 200 keywords.

To develop the second dataset, metrics from *Google Trends* on search trends in the search engine were obtained; these data illustrate how often a search for a particular term is performed in various regions and countries of the world and in several languages. *Google Trends* helps to detect moments when the popularity of a term increases and provides the average value of the relative interest in a selected time range. In this phase of data collection, the terms “climate change”, “global warming”, “climate emergency” and “greenhouse effect” were again applied for the period between April 2016 and March 2021 (both inclusive). For each term, the data for the weekly trends were downloaded for the entire period analyzed. The values were transformed to a scale from 0 to 100, where 100 indicated the week with the highest frequency of searches in proportion to the total number of queries; 50 and 0 indicated the weeks where the popularity of the term was half in relation to the maximum value or in which there were not enough data for the calculation, respectively.

For this study, we also obtained information on a series of variables related to the searches to find potential associations between variables. Thus, the following were considered: (a) the volume of news in the media between April 2016 and March 2021 (for this variable, the data related to the daily number of news stories appearing in RTVE newscasts and the Spanish public television and radio station, using the Civio search engine https://verba.civio.es, were considered), from which two values were established, i.e., (1) weeks during which the number of news items on CC was below the total average and (2) weeks during which the number of news items on CC was above the total average; (b) extreme meteorological phenomena that occurred weekly between April 2016 and March 2021; these data were obtained from monthly reports released by the Annual Climatological Summary of the State Meteorological Agency (AEMET, in Spanish) from 2016 to 2020 and for the year 2021 (https://bit.ly/3g0JmgE); (c) events of great importance on CC between April 2016 and March 2021, such as world summits on CC and COPs promoted annually by the United Nations Framework Convention on Climate Change (UNFCCC https://bit.ly/3fPVOQc) and climate-related demonstrations and strikes promoted by the student movement *Fridays for Future*—España (https://juventudxclima.es/); and (d) reports by the IPCC (https://www.ipcc.ch/reports).

### Data processing and analysis

Data processing and analysis were carried out using the statistical analysis program SPSS V.22, and the following statistics were used: frequency analysis, Student’s *t*-test to compare means, and one-way ANOVA.

## Results

### Keywords used to search for information related to CC and search volume in a 12-month period

In relation to the first of the objectives of this research (Identify, analyze, and classify the descriptors used in organic searches about CC between April 2020 and March 2021), from the list of 200 keywords generated with *SEMrush*, monthly search volumes averages varied among the 33,100 organic searches for the keyword “climate change” (see Table 1, which presents the 20 keywords with the most monthly searches, on average), and 100 searches that accumulated 110 of the keywords (the analysis limit an average equal to or >100 monthly searches for the period between April 2020 and March 2021). The total volume of monthly searches for the 200 analyzed terms was 146,830 searches. These data, converted to an annual average, indicate that during the period considered, more than 1,750,000 searches were carried out in Spain with the 200 descriptors analyzed. As seen (Table 1), the terms “climate change”, “greenhouse effect” and “global warming” had the highest search frequency throughout the year, with greater relevance of the term “climate change” among these first 20 keywords. However, the term “climate emergency” is also relevant, occupying position 11 in the ranking.

**Table 1:** List of the 20 keywords with the highest monthly search volume.

Keywords	Average volume of monthly searches
climate change	33,100
greenhouse effect	18,100
global warming	12,100
what is climate change	5400
what is the greenhouse effect	4400
consequences of climate change	4400
causes of climate change	3600
greenhouse gases	2900
what is global warming	2400
climate change definition	1600
climate emergency	1600
greenhouse gases	1600
causes of global warming	1600
consequences of global warming	1600
consequences of the greenhouse effect	1600
climate change news	1600
climate change drawings	1300
climate change for children	1300
greenhouse effect for children	1300
photos of climate change	1300
Total	34,750

**Fig. 1:**
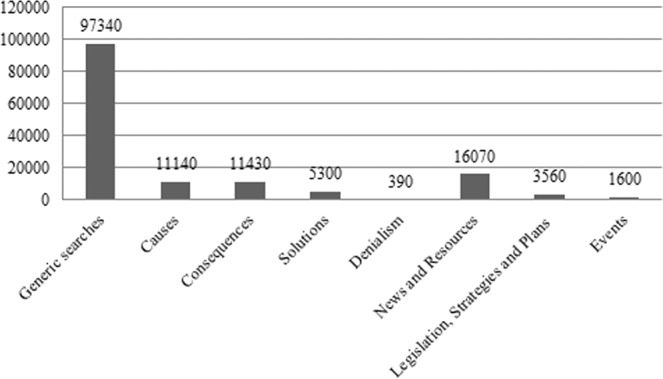
Monthly average of organic searches in Spain for each thematic category (April 2020/March 2021). Please note that eight search categories were determined.

The 200 keywords were coded into eight categories, classified, and counted based on the types of searches performed. As seen in Fig. 1, the greatest volume of searches was focused on locating general information about CC (for example, “what is climate change”, “climate change definition”, “climate change in Spain”, and “global warming definition”). Second were searches aimed at finding news and resources, using terms such as “global warming news,” “climate change for children,” and “images of climate change”. Third (with very even figures) were searches for the causes and consequences of CC. The categories that generated a lower volume of searches were denialism (for example, “climate change does not exist” or “climate change denial”) and events related to the topic of interest (for example, “International Climate Change Day”, “climate change protest”, and “climate change summit”). The category legislation, strategies, and plans were relevant among these searches in the last year because of the interest in the Spanish CC law (in fact, most of the search terms of this category correspond to “climate change law”).

### Monthly search trends across a 12-month period

**Fig. 2:**
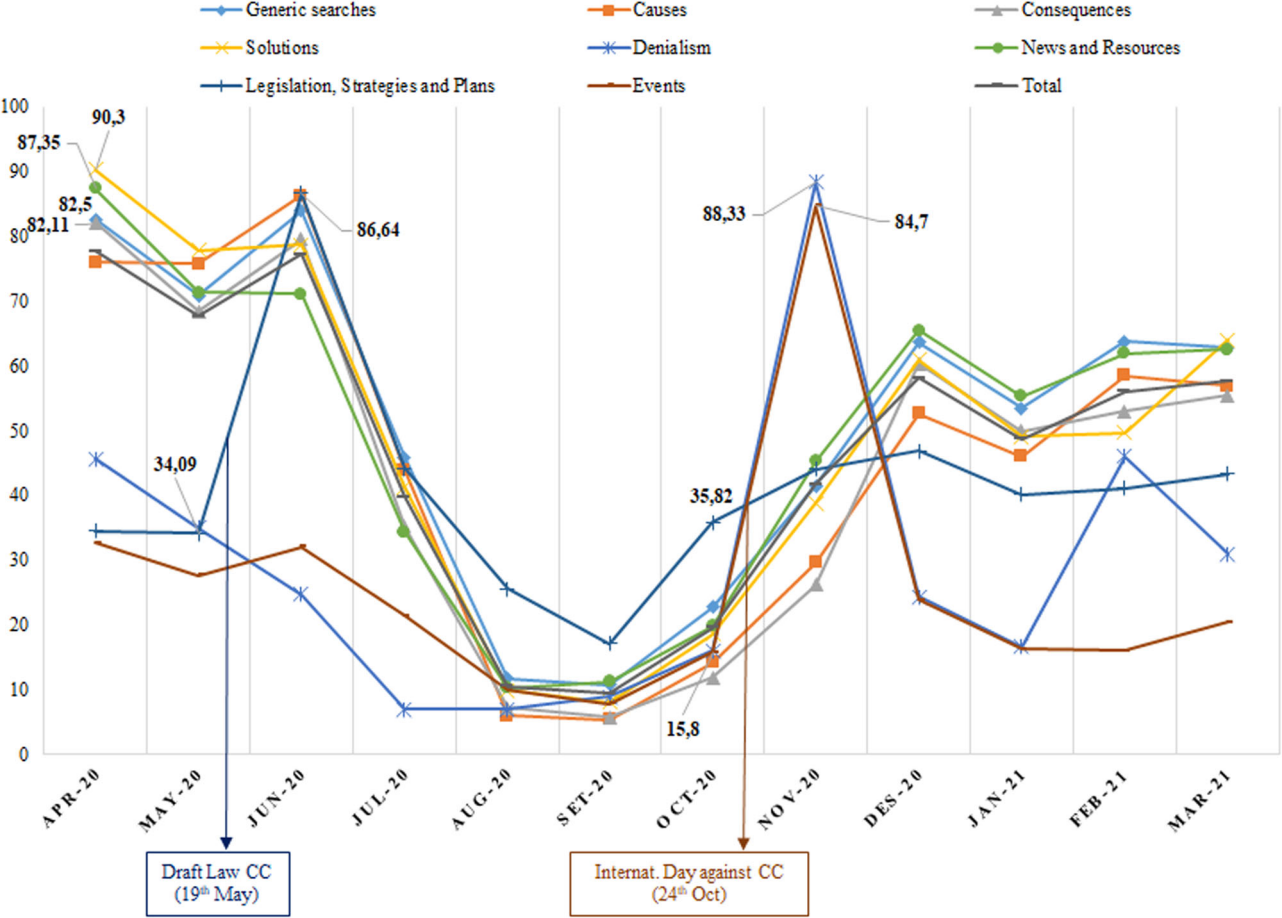
Monthly interest for the different search categories (April 2020/March 2021). Please note that the data legend in certain values of the categories highlights the variations in the searches. Dates of important events have also been highlighted in boxes.

Regarding the second research objective (Analyze the volume of activity and internet search trends for descriptors related to CC between April 2020 and March 2021), SEMrush provides metrics that measure trends in searches using each keyword based on the changes in the number of queries per month. The values range from 0 to 1, which we multiplied by 100. This parameter allowed establishing the monthly trends for searches using the categorized keywords in Spain for the 12 months analyzed (see Fig. 2). April, June, and November 2020 were the months with the highest search trends for the set of keywords analyzed, and the lowest trends occurred in August and September of that same year.

Variations in the level of interest in certain organic searches can be noted. For example, in April 2020, Solutions (90.3 points), News and Resources (87.35 points), Generic Searches (82.5 points), and Consequences (82.11 points) were popular categories. Subsequently, in May 2020, there was an increase of ~52 points in interest in the Legislation, Strategies, and Plans category (from 34.09 points on May 20 to 86.64 points on June 20). On May 19, 2020, the draft law for CC and energy transition in Spain was approved by the Council of Ministers. There was also a considerable increase in interest in the events category in October 2020, with an ~69-point difference between October (15.8) and November (84.7). On October 24, 2020, the International Day against CC was celebrated. It is striking that the interest related to the International Day against CC and the searches related to Denialism exhibited parallel increases (from ~16 points in October to 84.7 and 88.3 in November for Events and Denialism, respectively). Between January and February of 2021, there was also an increase in interest in Denialism, but not as substantial as that observed in October 2020.

### Annual search trends across the 5-year period

To achieve the third objective, focused on comparing the search trends for CC in Spain between April 2016 and March 2021, one-way ANOVA was performed. The existence of significant differences in the annual means of search interest in the four *keywords* was calculated using data from *Google Trends* (see Table 2). The results indicated that there were statistically significant differences in the interest in CC in the 5-year period for the keywords “climate change” (*p* = 0.000), “global warming” (*p* = 0.011), and “climate emergency” (*p* = 0.006). In general, beginning in 2016, there was an annual increase in searches using the four keywords analyzed. In 2019, searches peaked, with a significant difference with respect to the other years.

**Table 2:** Comparison of the annual means of search interest for the keywords analyzed (data obtained from Google Trends).

Keyword	Year	Mean search interest	Standard deviation	Standard deviation	Bilateral significance
Climate change	2016	6.1	4	0.655	0.000*
	2017	7.3	4	0.551	
	2018	8.3	4.2	0.585	
	2019	20.3	17.6	2.450	
	2020	13.6	6.5	0.903	
	2021	16.3	6.2	1.730	
	Total	11.6	10.4	0.644	
Global warming	2016	22.6	15.6	2.500	0.011*
	2017	22.2	13.0	1.787	
	2018	23.2	14.6	2.037	
	2019	31.9	18.4	2.557	
	2020	24.7	16.5	2.298	
	2021	30.9	10.3	2.879	
	Total	2.9	13.1	0.81	
Climate emergency	2016	0	0	0.000	0.006*
	2017	0.5	3.7	0.509	
	2018	0	0	0.000	
	2019	13.9	22.9	3.186	
	2020	12.1	20.7	2.871	
	2021	10.3	14.7	4.102	
	Total	16.7	17.9	1.10	
Greenhouse effect	2016	26.7	18.4	2.959	0,01
	2017	33.7	22.2	3.054	
	2018	36.1	21.7	3.011	
	2019	37.1	20.7	2.875	
	2020	44.2	28.8	3.995	
	2021	44.3	21.3	5.931	
	Total	36.4	23.2	1.439	
Summation	2016	55.5	32.5	5.215	0,07
	2017	63.9	33.4	4.599	
	2018	67.7	35.0	4.857	
	2019	103.4	59.8	8.299	
	2020	94.3	57.7	8.005	
	2021	102.0	34.5	9.580	
	Total	79.2	48.7	3.019	

*Significant at *p* < 0.05.

### Relationship between internet searches for CC and media coverage of CC between 2016 and 2021

Regarding the relationship between the volume of news appearing in RTVE newscasts on topics related to CC and internet searches for this environmental problem (the fourth objective of this study), the results obtained for the four keywords analyzed indicate that there are statistically significant differences in all cases; the greater the number of news items, the greater was the interest in searching for the concepts analyzed: “climate change” (*p* = 0.000), “global warming” (*p* = 0.000), “climate emergency” (*p* = 0.000) and “greenhouse effect” (*p* = 0.018) (Table 3).

**Table 3:** Relationship between the media coverage of climate change and internet searches using the keywords analyzed (data were obtained from Google Trends).

Keyword	News	Mean search interest	Standard deviation	Standard deviation	Bilateral significance
Climate change	Number of news stories less than the average	8.66	5.516	0.417	0.000*
	No. of news stories greater than the average	17.80	14.893	1.645	
Global warming	Number of news stories less than the average	2.49	8.966	0.678	0.000*
	No. of news stories greater than the average	13.23	22.760	2.513	
Climate emergency	Number of news stories less than the average	22.18	13.989	1.057	0.000*
	No. of news stories greater than the average	31.54	17.789	1.964	
Greenhouse effect	Number of news stories less than the average	33.74	23.652	1.788	0.018*
	No. of news stories greater than the average	41.00	20.816	2.299	
Total	Number of news stories less than the average	67.06	41.126	3.109	0.000*
	No. of news stories greater than the average	103.33	54.640	6.034	

*Significant at *p* < 0.05.

### Relationship between internet searches for CC and extreme meteorological phenomena that occurred between 2016 and 2021

The second of the variables studied for the fourth objective was the relationship between searches for CC and extreme meteorological phenomena, such as heat or cold waves, isolated high-level depression (DANA in its Spanish acronym), torrential rains, etc., that occurred during specific weeks in the last 5 years. That is, the aim was to analyze whether the occurrence of these phenomena influenced internet searches for CC. The statistical tests carried out indicated that there were only statistically significant differences between extreme meteorological phenomena and searches using the keyword “greenhouse effect” (*p* = 0.008) (Table 4).

**Table 4:** Relationship between the occurrence of external meteorological phenomena and internet searches using the keywords analyzed (data were obtained from Google Trends).

Keyword	Extreme weather events	Mean search interest	Standard deviation	Standard deviation	Bilateral significance
Climate change	Weeks with extreme events	12.16	11.206	0.781	0.500
	Weeks without extreme events	9.87	6.316	0.852	
Global warming	Weeks with extreme events	5.90	15.051	1.049	0.380
	Weeks without extreme events	5.55	17.255	2.327	
Climate emergency	Weeks with extreme events	26.03	16.106	1.122	0.550
	Weeks without extreme events	22.87	14.782	1.993	
Greenhouse effect	Weeks with extreme events	38.33	23.476	1.636	0.008*
	Weeks without extreme events	29.51	21.132	2.849	
Total	Weeks with extreme events	82.33	49.945	3.480	0.084
	Weeks without extreme events	67.80	42.564	5.739	

*Significant at *p* < 0.05.

### Internet searches and CC-related events between 2016 and 2021

The last of the relationships investigated in objective four was between internet searches for CC and “events.” This analysis included all weeks in which there was a climate demonstration or strike, a climate summit, or the release of an IPCC report. The results indicate a statistically significant relationship between CC-related events and an increase in internet searches using the keywords “climate change” (*p* = 0.000), “global warming” (*p* = 0.000), and “climate emergency” (*p* = 0.023); the association was not significant for “greenhouse effect” (*p* = 0.820) (Table 5).

**Table 5:** Relationship between events and internet searches using the keywords analyzed (data were obtained from Google Trends).

Keyword	Events on CC	Mean search interest	Standard deviation	Standard deviation	Bilateral significance
Climate change	Weeks with events	20.15	21.530	3.692	0.000*
	Weeks without events	10.41	6.660	0.442	
Global warming	Weeks with events	20.71	26.907	4.614	0.000*
	Weeks without events	3.60	11.518	0.764	
Climate emergency	Weeks with events	31.12	19.834	3.401	0.023*
	Weeks without events	24.51	15.043	0.998	
Greenhouse effect	Weeks with events	37.32	25.091	4.303	0.820
	Weeks without events	36.35	23.011	1.527	
Summation	Weeks with events	109.29	69.586	11.934	0.000*
	Weeks without events	74.78	43.281	2.873	

*Significant at *p* < 0.05.

## Discussions and conclusions

The results from this study reflect the public interest in CC in Spain in recent years based on internet search trends. The data obtained allowed us to identify which words and concepts are the most used by Google users in Spain to seek information related to CC and to determine the number of searches that this problem generates, which is substantial because the average monthly number of searches between April 2020 and March 2021 was 34,750. The first noteworthy observation regarding the search trends is how interest in CC increased in the last 5 years (2016–2021) among the Spanish population. A trend was also observed between 2017 and 2019 in relation to concerns about CC (European Commission, 2019). The year 2019 was a milestone regarding the number of searches. This trend can be explained by the student climate movement that began worldwide in 2019, with repercussions in Spain and with two school climate strikes (March and September 2019). In addition, in 2019, the COP25 was held in Spain. However, this interest decreased with the emergence of the pandemic in 2020 and 2021. This was the time of global lockdowns, which banned demonstrations and delayed summits such as COP26. What is more, COVID-19 increased the need for public information about the coronavirus (Bento et al., 2020; Vijay et al., 2021), reducing the space and interest for information on other issues. For example, compared to 2019, in 2020, there was a decrease of 23% in the appearance of news about CC in media worldwide (Fernández-Reyes and Jiménez-Gómez, 2021).

The monthly search trends for 2020 and 2021 in Spain fluctuated, coinciding with various key events. For example, in May 2020, there was an increase in searches for terms in the Legislation, Strategies, and Plans category. This likely occurred because a CC and energy transition bill was approved by the Council of Ministers of Spain (May 19, 2020), generating great interest among the Spanish population. In October 2020, there was also a considerable increase in searches using terms in the Events category, probably because October 24 was the International Day against CC. What is more, this data, compared with those from the Media and Climate Change Observatory—ECCO (Fernández-Reyes and Jiménez-Gómez, 2021) on media coverage of CC indicates an increase in the number of publications in the main Spanish newspapers in October and November 2020 compared to the two previous months. This fact could also explain the surge in Internet searches by the Spanish population on this event, given the positive relationship shown in this study between these searches and media coverage of the CC. Searches related to denialism peaked in some months (October and November 2020 and January and February 2021); however, this does not necessarily indicate that Spanish society doubts the existence of CC. In our study, there was a low number of organic searches using terms in this category (see Fig. 1), and the demoscopy in this sense indicated that such beliefs are residual and have gradually decreased since 2010, from 8.5 points (Meira et al., 2011) to 3.5 points (Ideara, 2021). Relationships between internet searches for CC and media coverage of CC, the occurrence of extreme weather phenomena, and CC-related events were analyzed in this study to establish statistically significant differences among the searches using the four keywords analyzed. The results obtained indicate that the increase in news stories about CC in the media implies an increase in interest by the population in information on the internet about CC, a finding also found in previous studies of written press for Spain (Fernández-Reyes, 2015). However, it is striking, and to some extent worrisome, how this positive relationship is also established with the keyword “greenhouse effect.” This is not an isolated fact; the confusion and interrelation between CC, the greenhouse effect, and the hole in the ozone layer are common in the existing scientific literature (Bozdogan, 2011). Thus, as stated by Meira (2007), this erroneous conceptualization has become part of the common culture, and the “greenhouse effect” is extensively used as a synonym for CC.

Unlike the results obtained in previous studies (Lang, 2014; Lang and Ryder, 2016), the occurrence of extreme weather events did not increase the interest of the Spanish population in CC, at least not for three of the four keywords analyzed. This trend could indicate, therefore, that the Spanish population considers these phenomena as isolated events and not as consequences of CC or as having no influence on their interest or concern for this problem. This variation in searches on CC seems to coincide, precisely, with the data reported by studies on the perception of CC in Spain: in 2019, 66.5% of the Spanish population considered that CC was being given less importance than it should (Valdor et al., 2019), and in 2020, this percentage increased by 6.5 points (Ideara, 2021). However, this argument is controversial because this relationship has been observed with searches using the keyword “greenhouse effect.” Therefore, we understand that the erroneous relationship between CC and the greenhouse effect continues to be perpetuated throughout society.

The occurrence of CC-related events, the last of the variables analyzed in this study, influenced the interest in CC among the Spanish population, especially during weeks in which these events recur. Previous research in this regard has yielded similar results: positive associations between interest in CC and student climate movements (Mavrodieva et al., 2019) or international climate meetings (Hartwell et al., 2020; Fernández-Reyes, 2015). Notably, this relationship is observed for the keywords “climate change,” “global warming” and “climate emergency” but not for “greenhouse effect.” This finding may be due not only to the use of these words during these events, underscoring the importance and gravity of the problem, but also to the fact that the language and concepts used at such events invoke a greater degree of climate literacy.

In light of the results obtained in this study, it could be concluded that the interest of the Spanish population in CC has increased on the internet in recent years and that some of the variables that may influence this interest are media coverage of CC, CC-related events and the social pressure exerted by climate-related social movements. The interest of citizens in CC could indicate the degree of social concern for this problem and even serve as a factor of public pressure for effective solutions against CC. However, we cannot determine that greater interest in CC is necessarily related to support for the cause. In fact, people’s support for the cause depends more on their values, ideologies, and political orientations than on other factors (Hornsey et al., 2016). Nevertheless, this does not obviate the need for the public to be properly informed about a problem such important as is CC. The results obtained, especially if we consider the errors of the concept identified in this study, do not allow us to affirm that searches for this information imply correct climate literacy, even considering the importance of this literacy for making informed decisions regarding the problem.

The Spanish population’s knowledge of the causes of the problem is not in line with the science of CC, and conversely, much of it does not demand more information on the subject (Ideara, 2021). To improve climate literacy, we believe that it is important to strategically communicate information related to CC because a better-informed public opinion will not perpetuate erroneous ideas regarding CC. Therefore, we believe that interest in topics related to CC should be encouraged and that evidence-based results managed by the scientific community and CC professionals should be presented to the public as accessible, attractive, and data-driven information. We consider fundamental, for example, the Decalogue elaborated by the Spanish Communication of Climate Change Observatory (OCCC, acronym in Spanish), in collaboration with communication professionals (Ecodes, 2022), which underlines actions as necessary from the journalistic profession as communicating the information of the IPCC reports or disseminating the specific concepts and terms necessary for a correct understanding of the problem. But we also appeal to the need for the development of specific websites that facilitate citizens’ access to information related to CC, as stated in the Spanish legislation on CC (Law 7/2021). However, it is not only access to information that is important but also the message that is disseminated with this information. We also believe in the importance of CC communication which equally influences the dissemination of the causes, impacts, and solutions of CC. For this, new ways of communicating the results of studies should be found, for example, through collaboration with key actors for scientific dissemination, such as environmental journalists, environmental disseminators, and environmental educators.

In any case, we would like to point out that the results presented in this study are not without certain limitations. Firstly, because they are not generalizable, as this is a study in a country where it is estimated that 96% of households have access to the Internet (Instituto Nacional de Estadística [INE], 2021). Secondly, because the sources used to compare certain variables, such as media coverage or the occurrence of extreme weather events, are national in scope.

## Data Availability

Due to its proprietary nature, supporting data cannot be made openly available. Derived data supporting the findings of this study are available from the corresponding author upon reasonable request.

## Footnotes

1 https://juventudxclima.es/

2 https://teachersforfuturespain.org/

3 https://madresporelclima.org/
